# Unsilencing a cryptic xylose metabolic pathway in *Rhodococcus jostii* RHA1 for efficient lipid production from lignocellulosic biomass

**DOI:** 10.1186/s13036-025-00503-1

**Published:** 2025-04-14

**Authors:** Miguel G. Acedos, Isabel De la Torre, Jorge Barriuso, José L. García

**Affiliations:** 1 Department of Biotechnology, Centro de Investigaciones Biológicas Margarita Salas, Consejo Superior de Investigaciones Científicas (CSIC), Madrid, Spain; 2 Advanced Biofuels and Bioproducts Unit, Department of Energy, Centro de Investigaciones Energéticas, Medioambientales y Tecnológicas (CIEMAT), Madrid, Spain

**Keywords:** Adaptive laboratory evolution, Lignocellulose, Xylose, Lipids, Biofuels, *Rhodococcus*

## Abstract

*Rhodococcus jostii* RHA1 is an oleaginous bacterium that has attracted considerable attention due to its capacity to use different carbon sources to accumulate significant levels of triacylglycerols that might be converted into biofuels. However, this strain cannot transform xylose into lipids reducing its potential when growing on saccharified lignocellulosic biomass. In this work, we demonstrate that wild type *R. jostii* RHA1 can be evolved by adaptive laboratory evolution (ALE) to metabolize xylose without engineering heterologous metabolic pathways in the host. We have generated a phenotypically adapted ALE-xyl strain able to use xylose as the sole carbon and energy source more efficiently that an engineered recombinant strain harbouring heterologous *xylA* and *xylB* genes encoding a xylose isomerase metabolic pathway. The *R. jostii* RHA1 ALE-xyl strain accumulates lipids very efficiently using xylose as substrate, but even more importantly it can consume glucose and xylose at the same time. Transcriptomic analyses of ALE-xyl strain growing with glucose or xylose revealed the existence of a silent pentose metabolizing operon that is overexpressed in the presence of xylose. The detection of a xylose reductase activity together with the presence of xylitol in the cytoplasm of ALE-xyl strain suggests that xylose is consumed by a reductase pathway. This study demonstrates that, in cases where a clear phenotypic selection method is available, ALE can be used to improve very efficiently industrial microbial strains without using genetic engineering tools. Strategies focused to exploit the silent phenotypic flexibility of microorganisms to metabolize different carbon sources are powerful tools for the production of microbial value-added products using saccharified lignocellulosic wastes.

**Supplementary Information:**

The online version contains supplementary material available at 10.1186/s13036-025-00503-1.

## Highlights

*Rhodococcus jostii* RHA1 is able to use xylose as sole carbon and energy source by activating a silent pentose degrading operon after a short adaptative selection process.

The xylose degrading capacity acquired by *Rhodococcus jostii* RHA1 is reversible suggesting that it is due to the phenotypic flexibility of this microorganism.

The evolved *Rhodococcus jostii* RHA1 strain simultaneously consumes glucose and xylose.

The evolved *Rhodococcus jostii* RHA1 strain is able to efficiently use xylose to produce lipids.

**Supplementary Information:**

The online version contains supplementary material available at 10.1186/s13036-025-00503-1.

## Graphical abstract

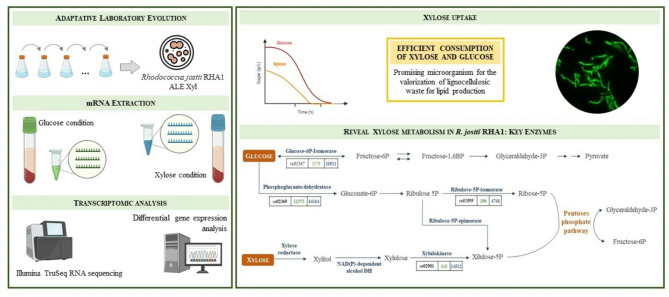

## Introduction

Hemicellulose represents a group of complex polysaccharides that is the second largest category of plant cell wall polysaccharides in nature after cellulose. It has a heterogeneous chemical structure, composed of glucose, xylose, and other sugars in less proportion such as L-arabinose. Xylose derived from hemicellulose can account for 18–30% of the monosaccharides present in lignocellulose hydrolysates, and therefore, it is considered a relevant renewable resource [1]. However, only a limited number of bacteria are able to metabolize xylose in order to produce different bio-based products as biofuels and chemicals [2].

**Fig. 1:**
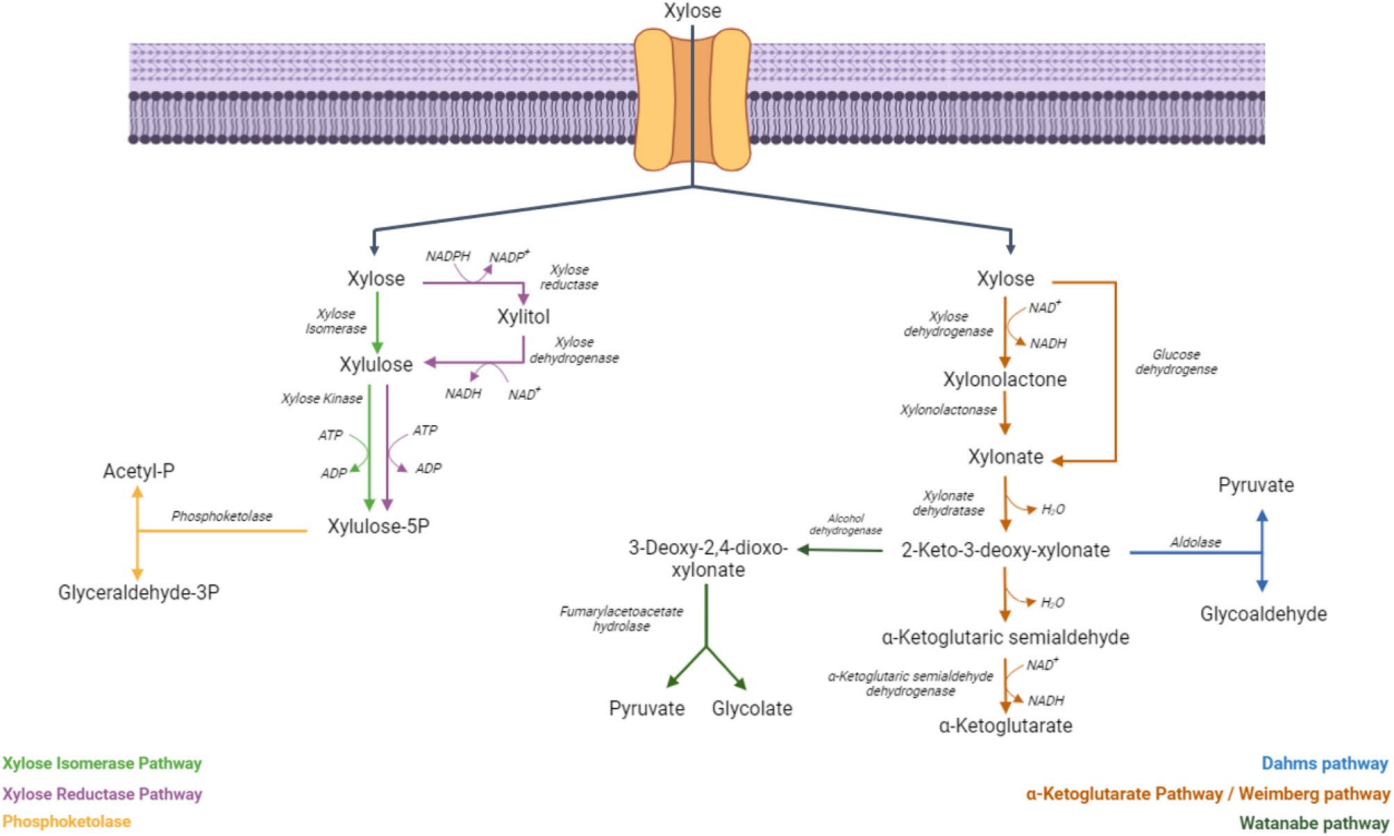
Natural xylose metabolic pathway described in different microorganisms

Microorganisms have evolved different metabolic pathways to assimilate xylose [1]. The isomerase pathway is the most commonly present in bacteria (e.g. *Escherichia coli*,* Bacillus subtilis*), where xylose is initially converted to xylulose by xylose isomerase (XylA), then xylulose is phosphorylated by xylulokinase (XylB) to xylulose-5-phosphate which is metabolized by the pentose phosphate pathway or cleaved into acetyl phosphate and glyceraldehyde-3-phosphate by a phosphoketolase [1] (Fig. 1). In contrast, most yeasts and mycelial fungi (e.g., *Saccharomyces cerevisiae*,* Candida spp.*) metabolize xylose through the reductase pathway, where xylose is converted into xylitol and subsequently into xylulose which is further phosphorylated by a xylulose kinase. Xylose can also be metabolized by a non-phosphorylative metabolic pathway, called the Weimberg pathway, discovered a few years ago in *Caulobacter crescentus*, which uses α-ketoglutarate as a key intermediate [3] (Fig. 1). Moreover, xylose can be converted into pyruvate and glycolaldehyde, using 2-keto-3-deoxy-xylonate as intermediary, through the Dahms pathway [4, 5] (Fig. 1). Finally [6], described a novel non-phosphorylative pathway of xylose metabolism in *Herbaspirillum huttiense* that renders pyruvate and glycolate (Fig. 1).

Xylose metabolism also requires xylose uptake and a regulatory systems [1]. Xylose transporters have been identified in different microorganisms within the ATP-binding cassette (ABC) family (e.g., XylFGH), or within the major facilitator superfamily (MFS) (e.g., XylE, AraE, or XylT) [1]. In many microorganisms, xylose metabolism is severely repressed by glucose through the carbon catabolite repression complex CRP-cAMP or other specific and pleiotropic regulators (e.g., XylR, CcpA) [1, 7].

The isomerase metabolic pathway to assimilate xylose has been reconstructed in bacteria with industrial interest such as *Corynebacterium glutamicum*, *Rhodococcus opacus*, *Pseudomonas putid*a or *Zymomonas mobilis*. For this purpose, *xylA* gene encoding a xylose isomerase has been used alone or in combination with *xylB* gene encoding a xylulokinase [8–18]. In addition, the Weimberg pathway for xylose assimilation has been also engineered in *C. glutamicum* [19].

Among bacteria with industrial interest are the oleaginous species, *Rhodococcus jostii* RHA1 and *R. opacus* PD630 which have attracted considerable attention due to its capability to utilize different compounds as carbon sources, and to accumulate significant levels of triacylglycerols as intracellularly microbial oils that can be utilized for biofuels [20]. Since these strains cannot use xylose or arabinose as carbon sources they have been engineered to use these sugars to produce lipid from saccharified lignocellulosic materials [10, 14, 21]. Interestingly, in *R. opacus* and *R. jostii* it has been demonstrated that only the expression of a heterologous *xylA* gene is necessary to facilitate their growth on xylose, since these strains contain two *xylB* genes encoding enzymes with xylulokinase activity [10, 14].

Given that *R. jostii* RHA1 has a xylulokinase activity, we hypothesize that perhaps this strain might have a silenced capacity to metabolize xylose that could be revealed by performing an adaptive laboratory evolution (ALE). This approach uses Darwinian rules for artificial selection of desired traits in microorganisms [22]. Although ALE has been successfully used to improve the production of lipids in *Rhodococcus* [23], it has not been used so far to evolve the capacity to metabolize xylose without the assistance of genetic engineering techniques.

The aim of this work was to use ALE to confer the wild type *R. jostii* RHA1 strain the capacity to use simultaneously glucose and xylose as carbon sources to produce lipids. In addition, we have compared its performance with an engineered version of *R. jostii* RHA1 carrying heterologous *xylA* and *xylB* genes without further improvements. Moreover, transcriptomic analyses revealed that RHA1 contains a silent operon most probably involved in the metabolism of pentose sugars that can be unsilenced by ALE to allow the growth of this strain and the production of lipids on xylose.

## Materials and methods

### Strains, media and culture conditions

*R. jostii* RHA1 strain (kindly provided by Dr. J. M. Navarro-Llorens from the University Complutense of Madrid) was used for this work and the *Escherichia coli* DH5α strain (Thermo Fisher Scientific) was used to clone the *xylAB-atf1* artificial synthetic operon in pNVS shuttle vector. All strains used in this work has been maintained in glycerol 20% and stored at -80 ºC.

*Rhodococcus* strains were grown at 30 °C on minimal medium W containing glucose or xylose as carbon sources. The composition of the W medium (per litre) was: 0.85 g KH_2_PO_4_, 4.90 g Na_2_HPO_4_ 12H_2_O, 0.50 g (NH_4_)_2_SO_4_, 0.10 g MgSO_4_ 7H_2_O, 9.50 mg FeSO_4_ 7H_2_O, 10.75 mg MgO, 2.00 mg CaCO_3_, 1.44 mg ZnSO_4_ 7H_2_O, 1.12 mg MnSO_4_ 4H_2_O, 0.25 mg CuSO_4_ 5 H_2_O, 0.28 mg CoSO_4_ 7 H_2_O, 0.06 mg H_3_BO_4_ and 5.13 × 10^− 2^ mL concentrated HCl. Stock solutions of the sugars, including glucose, xylose and arabinose were prepared at 30 g/L and were sterilized with a 0.22 μm pore size filter before adding to the autoclaved medium. Tests with sugar mixtures were carried out at concentrations similar to those obtained in an enzymatic hydrolysis of corn stover sample (glucose: xylose: arabinose of 20:6:1) in our laboratory (data not shown). Thus, a mixture of 20 g/L of glucose, 6 g/L of xylose and 1 g/L of arabinose were used for fermentation experiments.

To accumulate lipids, the strains were grown under nitrogen-limited conditions. For the preparation of inocula, *Rhodococcus* cells were grown in LB medium and harvested by centrifugation at 8,000 *× g* and 4 °C for 10 min. The pellets were washed twice with W medium without sugar and nitrogen. After washing, the cells were inoculated into 100 mL of W medium with 30 g/L glucose or xylose and 0.5 g/L (NH_4_)_2_SO_4_ as nitrogen source. The culture was incubated at 30 °C and 200 rpm in a 500 mL flask. The cells, cultured for 72 h, were harvested and washed before lipid analysis.

### ALE methodology

To evolve *R. jostii* RHA1, the strain was firstly grown in LB plates, then 20 colonies were selected randomly and inoculated in 20 mL of W minimal medium containing 30 g/L xylose as only carbon source and cells were cultured at 30 °C and 200 rpm in a 100 mL flask. After 1 week we observed a slight increase in the DO of the culture and then, cells were harvested by centrifugation and resuspend in fresh W medium plus xylose and cultured again in the same conditions. After 9 subcultures of one week each we observed a progressive increase in the OD of the subcultures suggesting that the cells able to efficiently metabolize xylose were increasing. Then, cells of the last subculture which showed a high DO were plated in W agar medium plus xylose and several clones that grew well in the plates were isolated. One of the isolated clones was named ALE-xyl strain and stored in glycerol 20% at -80 ºC. A scheme of the process is shown in Figure S1.

### Construction of the Recombinant strain

The synthetic operon, named *xylAB-atf1*, was designed to express the following genes: *xylA* (xylose isomerase) and *xylB* (xylulokinase) from *Streptomyces lividans* TK23, and *atf1* (diglyceride acyltransferase) from *R. opacus* PD630 [14, 21], were cloned in the *E. coli-Rhodococcus* pNVS shuttle vector under the control of *Ptac* promoter with kanamycin resistance. The *atf1* gene was included in order to improve the production of lipids [21]. To achieve optimal translation of the mRNA a consensus Shine Dalgarno sequence (AGGAGG) was added upstream each gene at 6 bp from the respective start codons. Codon usage was adapted to *Rhodococcus* using the Optimizer program (Genescript). The synthetic operon *xylAB-atf1*, synthesized by Genescript, was cloned into the pNVs vector using the restriction enzymes *Spe*I and *Pac*I (Fig. S2).

Competent *E. coli* DH5α cells were prepared using CaCl_2_ and subjected to transformation through the heat shock procedure [24]. *Rhodococcus* cells were cultivated in LB medium until reaching an optical density of 0.5–0.6, then were harvested, washed with a solution of 10% glycerol, and subsequently electroporated with the plasmid (using conditions of 400 Ω, 25 µF, and 2.5 kV). Immediately following electroporation, cells were extracted from the cuvette and cultivated in 1 mL of LB medium in sterile 10 mL plastic tubes for 6 h at 30 ºC, without agitation. Finally, the cells were plated on LB agar plates supplemented with 150 µg/mL of kanamycin.

### Transcriptomic analysis

The ALE-xyl strain was cultured in LB medium as a pre-inoculum. This culture was used to inoculate 100 mL flasks containing 20 mL of W minimal medium supplemented with glucose (20 g/L) or xylose (20 g/L). The culture was started with an OD_600_ of 0.5 and incubated at 30 ºC and 180 rpm in an orbital shaker. After 40 h of culture, i.e., at the middle exponential growth phase (OD_600_ = 3.5), cells were centrifuged 5 min at 8,000 rpm (Eppendorf Centrifuge 5810R) and 4 ºC. The harvested cells were washed twice with saline solution (0.9% NaCl) by centrifugation 5 min at 8,000 rpm (Eppendorf Centrifuge 5810R) and 4 ºC. The biomass was stored at -80 ºC until processing. All assays were performed in three independent biological replicates as described [25]. For RNA extraction, the cell pellet was resuspended in 1 mL of a solution containing 1% SDS (v/v), 160 mM EDTA, and 50 mg/mL lysozyme. This mixture was transferred to a 15 mL Falcon tube with sterilized glass beads and left at room temperature for 5 min to allow the lysozyme to break down the cells. After, 200 µL of phenol-chloroform-isoamyl alcohol (ROTH) were added and the mixture was vortexed. Then, 800 µL of RLT buffer (Qiagen) with β-mercaptoethanol (100:1) were added and the mixture was incubated on ice for 10 min. Thereafter, the mixture underwent three 30-second vortexing cycles followed by incubation on ice. Subsequently, 1.4 mL of phenol-chloroform-isoamyl alcohol were added. The Falcon tubes were centrifuged for 15 min, and the aqueous phase was transferred to a new tube containing 700 µL of ethanol. Finally, the total RNA was purified using a RNeasy kit (Qiagen) following the instructions.

The three biological replicates of RNA from ALE-xyl strain grown with glucose or xylose, were sequenced by the Macrogen NGS Service10 that uses an Illumina TruSeq RNA library, providing a 6 GB/sample of 151 bp paired-end reads. Raw reads were trimmed and cleaned with Trimmomatic 0.39 in order to remove Illumina adapters and low-quality bases [26]. Trimmed reads were aligned to the *R. jostii* RHA1 genome (NCBI RefSeq assembly GCF_000014565.1) using the STAR 2.7.9a aligner [27], followed by the count of reads per annotated coding sequence (CDS) with HTSeq 0.11.3 [28]. Differential gene expression analysis between conditions and normalized counts were obtained with the R software DESeq2 package [29, 30]. A principal component analysis (PCA) plot was made to visualize gene expression pattern distances between replicates. Principal component scores were obtained with the plotPCA function of the package DESeq2 of R (Fig. S3A). To create the heat map, the similarity matrix shown in Figure S3B was obtained using the Euclidean distance and the cluster analysis was performed with the Ward minimum variance method.

### Xylose reductase activity

ALE-xyl strain was cultured in minimal medium W supplemented with 30 g/L of xylose at 30 °C and 200 rpm for 48 h. A culture sample of 10 mL was centrifuged at 16,000 × *g* for 10 min at 4 °C and the cell pellet was washed twice with 50 mM Tris-HCl buffer (pH 8.0). The washed cells were then resuspended in 3 mL of the same buffer. For cell disruption, 1 mL of the resuspended cells was transferred into 2 mL FastPrep plastic tube. The sample was mixed with 0.4 mL of glass beads and subjected to four grinding pulses of 40 s each in a FastPrep 24 homogenizer (MP Biomedicals™). The resulting lysate was centrifuged at 16,000 × g for 10 min at 4 °C. The supernatant obtained after centrifugation containing the soluble protein (about 4 mg/mL) was used to perform the enzymatic assay. Enzymatic activity was measured at 25 °C using a Shimadzu UV-1900i spectrophotometer, following the method described by Verduyn et al. (1985) [31]. Xylose reductase activity was assayed in 50 mM Tris-HCl buffer (pH 8.0) with 70 mM xylose as substrate and the crude protein extract. The reaction was initiated by the addition of 0.2 mM.

### Biomass and sugars determination

Growth was determined by measuring the optical density of the cultures at 600 nm in a spectrophotometer (Shimadzu UV-260). The concentration of glucose, xylose, xylitol and arabinose were determined by HPLC (Agilent Technologies, 1100 series) using a Rezex RHM-Monosaccharide-H^+^ 300 × 7.8 mm column (Phenomenex), and a refraction index detector (RID). A solution of 5 mM H_2_SO_4_ was employed as mobile phase at a flow rate of 0.5 mL/min. The column temperature was maintained at 80 ºC. The supernatant (crude extract) obtained for the enzymatic assays was used to detect intracellular metabolites by HPLC.

### Lipid quantification

The biomass was centrifuged at 3,800 rpm (Eppendorf Centrifuge 5810R) for 5 min and the pellet was lyophilized, then 20–30 mg were treated with 2 mL of a mixture of methanol/hydrochloric acid/chloroform (10:1:1) in a test tube sealed and gently shacked. The tube was heated at 90 ºC for 60 min to convert lipids into fatty acid methyl esters (FAMEs). FAMEs were extracted using hexane GC analysis grade. Heneicosanoic acid was employed as internal standard (10–20 µL at 10 mg/mL). The chromatographic analysis was conducted in an Agilent 7890 A GC-MS system, equipped with an Agilent 122–5731 column (30 m x 250 μm x 0.1 μm), with hydrogen as carrier gas. An injection volume of 1 µL was utilized with a split ratio of 10:1. The oven temperature initiated at 115 °C, followed by a ramp-up to 210 °C at a rate of 3 °C/min (35 min), and ultimately maintained at 280 °C for an additional minute. Identification and quantification of fatty acids were performed by comparing them to standard mixture FAMEs (Sigma). The fatty acid content was expressed as the percentage of fatty acids to cell dry weight (% CDW).

### Microscopy

The lipid bodies in the cells were observed after dyeing by adding Bodipy^®^ stain in the dark at 4 ºC for 10 min. Fluorescence images were taken with a Leica microscope DM4B (Wetzlar, Germany) at 493 nm excitation and 503 nm emission.

## Results

### Evolving *R. jostii* RHA1 to metabolize xylose

Based on the assumption that enzyme promiscuity and phenotypic flexibility of microorganisms shape adaptation to novel growth substrates [32], and the fact that *R. jostii* RHA1 natively has two *xylB* genes that can facilitate the consumption of xylose [14], we developed an ALE strategy to test if RHA1 can be evolved to efficiently metabolize xylose without expressing heterologous enzymes.

When we cultured *R. jostii* RHA1 in minimal media containing xylose as the sole carbon and energy source we observed a slight growth after 1 week of cultivation. After four passes, we were able to isolate one clonal strain named ALE-xyl able to grow very efficiently on minimal medium agar plates containing xylose. Figure S4 shows the growth of the wild type RHA1 and ALE-xyl strains in rich medium as well as in minimal media containing xylose as sole carbon and energy source. The identity of the ALE-xyl strain as a derivative of RHA1 was confirmed by its 16 S RNA sequence (data not shown).

To determine the stability of the xylose phenotype of the ALE-xyl strain, the isolated clone was cultured in rich medium and then transferred again to minimal medium containing xylose as sole carbon source. Figure S5 shows that ALE-xyl strain progressively loses the ability to grow on xylose when cultured in rich medium. The strain only maintains the ability to grow on xylose when cultured in minimal medium containing xylose suggesting that the capacity of ALE-xyl strain to metabolize this monosaccharide is most probably due to a reversible phenotypic adaptation.

### Xylose consumption by ALE-xyl strain

**Fig. 2:**
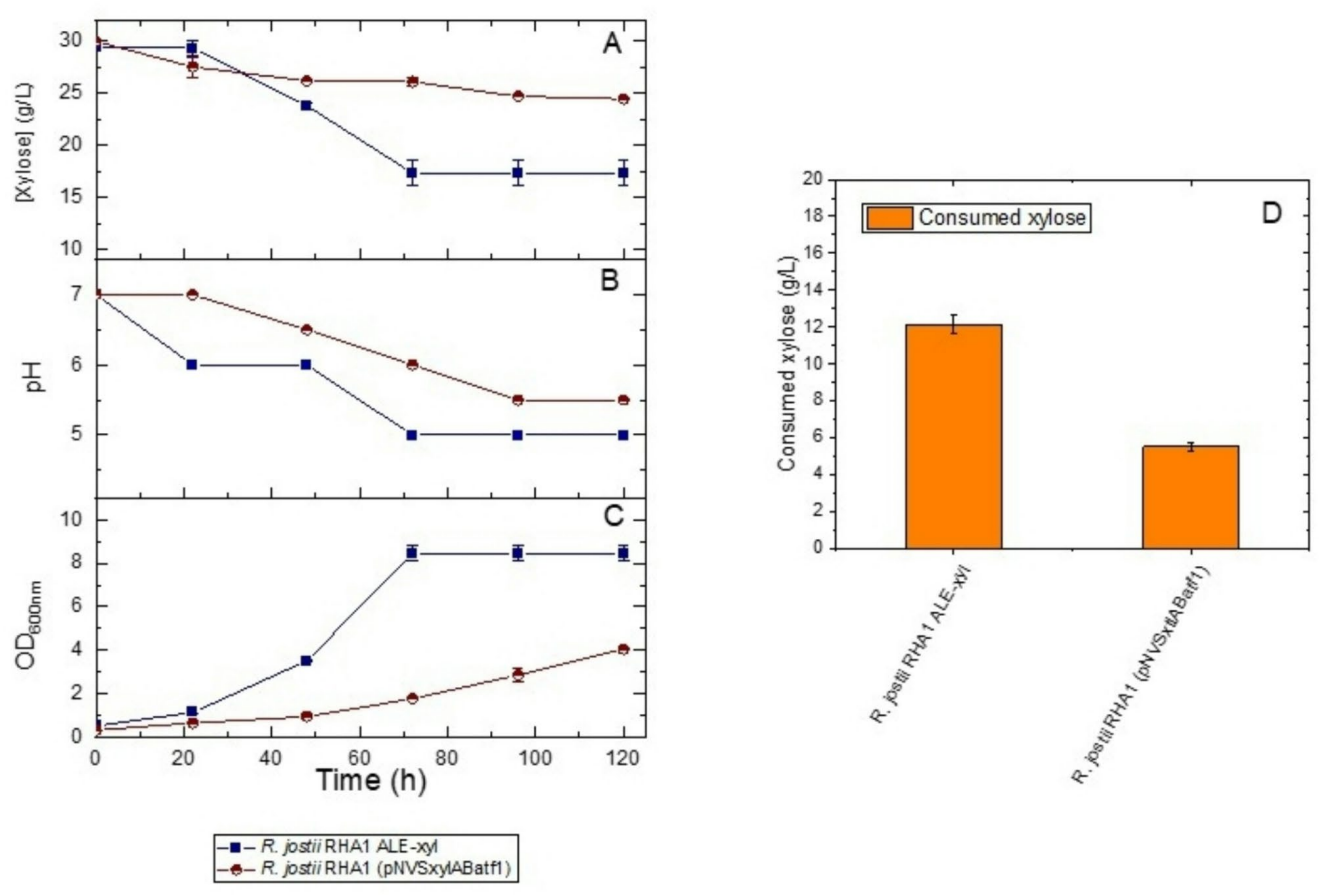
Time course of *R. jostii* RHA1 ALE-xyl and *R. jostii* RHA1 (pNVSxylABatf1) strains cultured in xylose. **(A)** Xylose consumption. **(B)** pH. **(C)** Optical density. **(D)** Total sugar consumption. The culture was carried out with xylose (30 g/L)

The growth on xylose of ALE-xyl strain was compared with that of the recombinant strain *R. jostii* RHA1 (pNVSxylABatf1), harbouring the xylose isomerase pathway, which was constructed in our lab to overproduce lipids using xylose as substrate. Figure 2 shows that both strains are able to grow when cultured in minimal W medium containing 30 g/L of xylose as the sole carbon and energy source. However, the xylose consumption by the ALE-xyl strain was higher than that observed in the recombinant strain which was not evolved by ALE.

We observed that when cells are cultured in flasks on xylose the pH of the medium decreases as far as xylose is consumed and when pH drops below 5 the strains stop growing (Fig. 2B), indicating that a strict control pH is critical to facilitate the complete consumption of xylose.

**Fig. 3:**
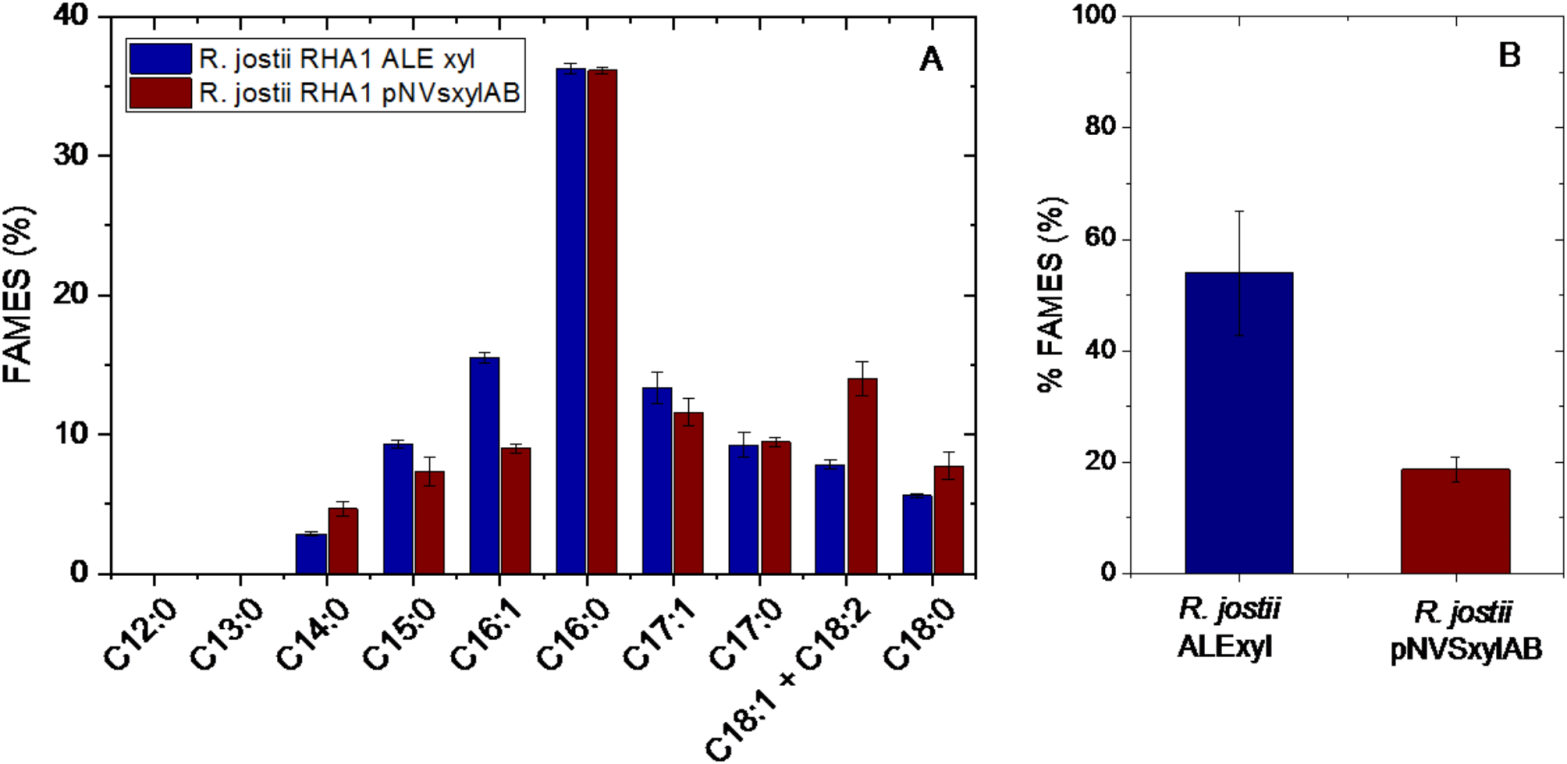
**(A)** Lipid content of *R. jostii* RHA1 ALE-xyl and *R. jostii* RHA1 (pNVSxylABatf1) strains cultured on media with xylose as sole carbon source. **(B)** FAMEs of ALE-xyl and *R. jostii* RHA1 (pNVSxylABatf1) strains cultured on media with xylose as sole carbon source

Figure 3 shows how ALE-xyl strain produces more lipids (54%) than the recombinant strain (19%) according to the percentage of FAMEs in relation to the total biomass. Moreover, FAMEs profile produced by both strains is very similar, being palmitic acid the most abundant fatty acid in both cases. Figure S6 shows microscopy images of lipids accumulated and produced by both strains.

### Growth of the ALE-xyl strain in a lignocellulosic-like hydrolysate

**Fig. 4:**
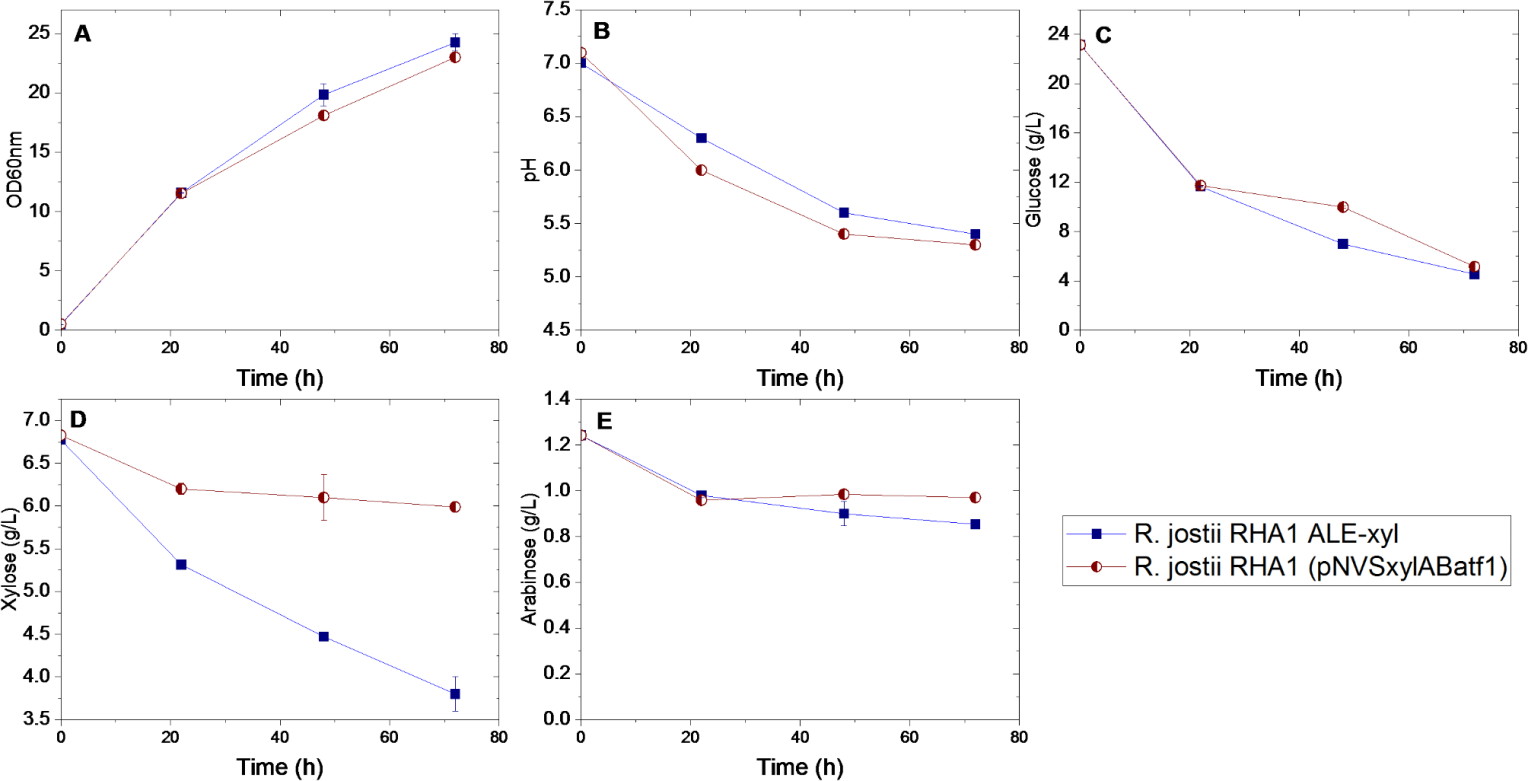
Time course of *R. jostii* RHA1 ALE-xyl and *R. jostii* RHA1 (pNVSxylABatf1) cells cultured in a mixture of sugars (glucose (20 g/L), xylose (6 g/L) and arabinose (1 g/L).): **(A)** Optical density at 600 nm; **(B)** pH; **(C)** glucose consumption; **(D)** xylose consumption; **(E)** arabinose consumption

To determine if ALE-xyl strain could be able to consume at the same time glucose and xylose in a lignocellulosic-like hydrolysate (glucose + xylose + arabinose), the strain was cultured in minimal W medium containing a synthetic mixture of glucose, xylose and arabinose mimicking the proportion that can be found in a saccharified extract of a corn stove prepared in our laboratory (glucose (27 g/L), xylose (7 g/L) and arabinose (1.2 g/L). Figure 4 shows how ALE-xyl is able to metabolize xylose in the presence of glucose. It is very interesting that in this evolved strain glucose does not repress the consumption of xylose. Moreover, as expected, arabinose is not metabolized by ALE-xyl, but interestingly, we have observed that it does not interfere with the consumption of glucose or xylose. The lipid production in the ALE-xyl strain in this medium was slightly lower (60%) than the percentage achieved when the strain was cultured using xylose as sole carbon and energy source.

### Transcriptomic analysis

**Fig. 5:**
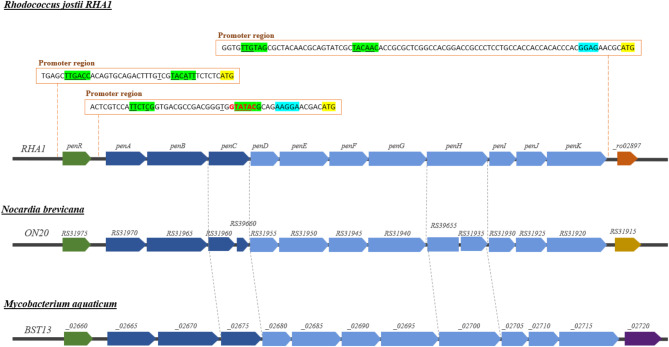
Scheme of the *pen* operon (*RHA1_ro02898 - RHA1_ro02909*) overexpressed in *R. jostii* RHA1 ALE-xyl strain growing in xylose. This operon is also present in microorganisms of *Actinobacteria* phylum

Once we had demonstrated that the ALE-xyl strain was capable of growing efficiently and producing lipids using xylose as the sole source of carbon and energy, we set out to investigate which genes could be responsible for the phenotypic adaptation that occurred in this strain. To this aim, we performed a comparative transcriptomic analysis of the ALE-xyl strain cultured in minimal medium containing glucose or xylose as a sole carbon and energy source (see supplementary material). These analyses revealed that xylose growing cells highly overexpressed the putative operon *RHA1_02898 – RHA1_ro02909* that we have named as *pen* operon (Fig. 5; Table 1). The function of this operon has not been previously described in *Rhodococcus* or in other microorganisms, but the annotated functionality that can be ascribed to the genes encoded in this operon suggests that it can be involved in the metabolism of pentose sugars. A systematic search on the genome data bases revealed that the same operon is present in several Rhodococci and in other closely related actinomycetes (Fig. 5).

**Table 1:** Genes from the *Pen* Operon (*RHA1_ro02898 - RHA1_ro02909*) overexpressed in *R. jostii* RHA1 ALE-xyl strain growing in xylose

Locus	Size (bp)	Name	log_2_ FoldChange	*p*-value
*RHA1_ro02897*	504	acyl-CoA thioesterase	4.53	2.73E-207
*RHA1_ro02898* *penK*	1455	NADP + dependent aldehyde dehydrogenase	4.24	1.29E-297
*RHA1_ro02899* *penJ*	759	ribose-5-phosphate isomerase. RpiA	4.60	2.76E-240
*RHA1_ro02900* *penI*	651	phosphoglycolate phosphatase	4.78	5.57E-229
*RHA1_ro02901* *penH*	1533	xylulokinase	4.48	0.00E + 00
*RHA1_ro02902* *penG*	1437	aldehyde dehydrogenase / succinate-semialdehyde dehydrogenase	4.46	0.00E + 00
*RHA1_ro02903* *penF*	951	D-3-phosphoglycerate dehydrogenase / 2-oxoglutarate reductase	5.50	0.00E + 00
*RHA1_ro02904* *penE*	1188	fumarylacetoacetate hydrolase family protein	5.33	0.00E + 00
*RHA1_ro02905* *penD*	717	SDR family oxidoreductase	5.72	0.00E + 00
*RHA1_ro02906* *penC*	1050	ABC transporter permease	5.67	0.00E + 00
*RHA1_ro02907* *penB*	1551	sugar ABC transporter ATP-binding protein	5.23	0.00E + 00
*RHA1_ro02908* *penA*	1047	substrate-binding domain-containing protein	5.41	0.00E + 00
*RHA1_ro02909* *penR*	705	FadR/GntR family transcriptional regulator	2.77	2.57E-167

Although the *RHA1_ro02897* gene encoding a putative acyl-CoA thioesterase located at the 3’-end of the *pen* operon is also overexpressed (Table 1), this gene does not seem to form part of the *pen* operon. The distance between the stop codon of the last gene of the operon and the start codon of the thioesterase encoding gene is too long (i.e., 194 bp), suggesting that this gene is expressed by an independent promoter (Fig. 5). In contrast, the distance within the stop and start codons of the contiguous genes contained in the *pen* operon ranges between 1 and 55 bp. The reason why this thioesterase is overexpressed, even when it is not apparently related to sugar metabolism, is unknown, nevertheless, we have detected a long palindromic region (*GTTGTA*GCG*TACAAC*) overlapping the boxes − 35 and − 10 of the gene (Fig. 5) that could be recognized as a consensus operator sequence by the PenR regulator.

Remarkably, the *pen* operon contains the *RHA1_ro02901* (*penH*) gene encoding the xylulokinase described by Xiong et al. (2012) to be functional on xylulose. However, the other xylulokinase (*RHA1_ro02812* gene) annotated in the genome of *R. jostii* RHA1 and also described by Xiong et al. (2012) as functional on xylulose was apparently expressed constitutively [14]. This finding can explain why *RHA1_ro02812* is more efficient in the wild type strain than *RHA1_ro02901* [14], because the last remain silent.

Remarkably, the *pen* operon encodes an ABC transporter (*penABC* genes) that is homologous to pentose transporters and that might facilitate the uptake of xylose. Although there are other annotated sugar transporters in the genome of RHA1, none of them are overexpressed in the presence of xylose. Even more the *RHA1_ro02804-RHA1_ro02808* genes encoding the only sugar ABC transporter that is overexpressed in glucose show expression levels far below those observed for the *penABC* genes (Table 2). Nevertheless, we cannot discard that other less expressed putative sugar transporters can contribute to the uptake of xylose.

**Table 2:** Sugars transporters expressed in *R. jostii* RHA1 ALE-xyl strain under glucose and xylose growth conditions

Locus	Name	Glucose (counts)	Xylose (counts)
*RHA1_ro02906*	ABC transporter permease	211	10,748
*RHA1_ro02907*	sugar ABC transporter ATP-binding protein	387	14,588.
*RHA1_ro02908*	substrate-binding domain-containing protein	489	20,804
*RHA1_ro02804*	sn-glycerol-3-phosphate ABC transporter ATP-binding protein UgpC	3800	502
*RHA1_ro02805*	carbohydrate ABC transporter permease	2434	445
*RHA1_ro02806*	sugar ABC transporter permease	2811	535
*RHA1_ro02807*	sugar ABC transporter substrate-binding protein	4668	988
*RHA1_ro02808*	sugar-binding domain-containing protein	1686	570

A FadR/GntR-like regulator, named as PenR, that is strongly expressed in the presence of xylose is located just upstream of the operon suggesting that the expression of the *pen* operon can be controlled by this regulator. An analysis of the promoter region suggests that most probably the mRNA of PenR does not have a Shine-Dalgarno sequence, because there is not a typical consensus sequence preceding its start codon and because the − 10 box of the putative promoter is located very close to the start codon. Therefore, this promoter could render a typical leaderless transcript frequently found in actinomycetes [33].

The in-silico analysis of the promoter region of the *pen* operon revealed that within the putative − 10 box there is a perfect palindromic GTATAC sequence that matches with the palindromic NyGTNxACNy consensus sequence proposed as operator for the GntR-like regulators [34] (Fig. 5). According to this observation we could propose that the PenR regulator might act as a repressor. Nevertheless, considering that PenR is overexpressed in ALE-xyl we cannot discard that this regulator can be an activator since GntR-like regulators can act either as repressors or activators.

Although the genome of *R. jostii* RHA1 contains many genes annotated as sugar isomerases none of these genes appear to be overexpressed in xylose and thus, we cannot propose that an isomerase could be responsible for metabolizing xylose through the typical isomerase pathway frequently found in bacteria. However, we cannot discard that the basal expression of one of these isomerases, that in general accept a wide range of substrates, can be enough to transform xylose into xylulose once the sugar is uptake by the ABC pentose transport system contained in the operon.

**Fig. 6:**
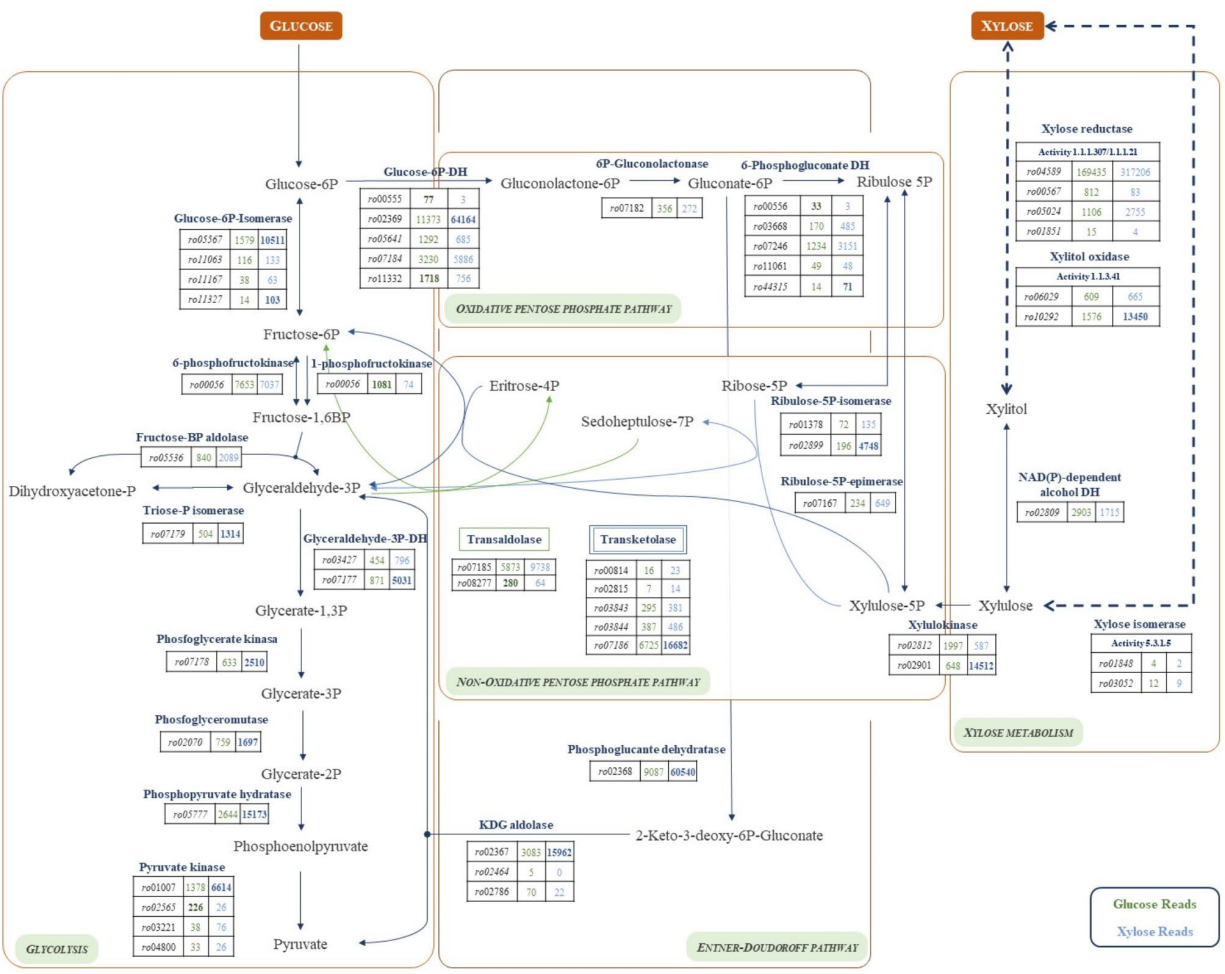
Expression levels of the genes involved in the first steps of the metabolism of glucose and xylose in *R. jostii* RHA1. The data are presented in counts

Figure 6 shows the differential expression of the annotated genes involved in the metabolism of hexoses and pentoses in *R. jostii* RHA1 when the ALE-xyl cells are cultured in glucose or xylose. These results strongly suggest that xylose might be metabolized in RHA1 strain through a reductase pathway as proposed for *Shewanella oneidensis* [35]. Interestingly, RHA1 contains at least three annotated putative aldolase/keto reductases encoded by the genes *RHA1_ro04589*,* RHA1_ro05024*, and *RHA1_ro00567* that can act as xylose reductases, and in fact some of them are constrictively and highly expressed in ALE-xyl, both in glucose and xylose containing media (See counts at Fig. 6). We have also observed a putative alcohol dehydrogenase encoded by the gene *RHA1_ro02809* that is highly expressed in glucose and xylose that might act as a xylitol/xylulose dehydrogenase (Fig. 6). Nevertheless, we cannot discard that other alcohol dehydrogenases could play this role as suggested by Sekar et al. (2016) [35].

Figure 6 shows that the glucose-6-P isomerase and the glucose-6-P reductase are overexpressed in the presence of xylose suggesting that the ribose-5-P required for the pentose pathway can be produced from the isomerization of ribulose-5-P generated by the oxidative reactions of the pentose pathway. A putative ribos-5-phosphate isomerase (*RHA1_ro02899* gene, *rpiA*-like) is encoded in the *pen* operon, reinforcing this hypothesis. Thus, we propose that half of the fructose-6-P produced by the non-oxidative reactions of the pentose pathway can be transformed by glucose-6-P isomerase into glucose-6-P that is further transformed by the glucose-6-P reductase into 6-phosphogluconolactone. Nevertheless, we cannot completely discard that ribose-5-P can be produced from xylulose-5-P through the serial action of ribulose-5-P epimerase that generates ribulose-5-P and the ribose-5-P isomerase that transforms the ribulose-5-P into ribose-5-P.

The transcriptomic analysis also revealed that the Entner-Doudoroff pathway is also functional in ALE-xyl either in glucose or xylose (Table S1). In *R*. *jostii* RHA1 the genes responsible for this pathway are organized in three operons within the *RHA1_ro02362- RHA1_ro02370* cluster. Interestingly, the *RHA1_ro02365* gene encoding a sugar MFS facilitator has been described as the main glucose transport system in RHA1 [36]. Considering that these genes are highly expressed in the presence of xylose we must assume that the regulation of these genes should be carried out by glucose-6-P or any other intermediate of the pathway. Interestingly, this cluster does not encode a gluconolactonase-6-P activity that is located elsewhere in the genome (*RHA1_ro07182*), but low expressed (Fig. 6), suggesting that gluconolactone-6-P can be also spontaneously transformed into gluconate-6-P as suggested by other authors [37].

Considering all alternative pathways, several types of metabolic reactions can take place in RHA1:

$$\begin{gathered}3{\text{ }}Xylose\, \to \,G3P\, + \,2\,F6P \hfill \\\,\,\,\,\,\,\,\,\,\,\,\,{\text{ }}\left( {through{\text{ }}epimerization - isomerization} \right) \hfill \\ \end{gathered} $$

$$\begin{gathered}2{\text{ }}Xylose \to \,C{O_2}\, + \,G3P\, + \,F6P\, \hfill \\\,\,\,\,\,\,\,\,\,\,\,\,\,\left( {through{\text{ }}oxidative{\text{ }}pentose{\text{ }}reactions} \right) \hfill \\ \end{gathered} $$

F6P → G3P and pyruvate (Entner-Doudoroff or Embden-Meyerhoff (glycolysis) pathways).

Surprisingly, we have observed that in the presence of xylose many of the S30 and S50 ribosomal genes are overexpressed when compared with the expression levels observed in glucose, suggesting that the metabolism of xylose requires the synthesis of a larger number of ribosomes (see Table S2). In this sense, ribosome abundance has been correlated with growth rate in *C. glutamicum* [38].

Finally, it is worth to mention that the *RHA1_ro04085-RHA1_ro04093* gene cluster that is annotated as a rhamnose metabolic cluster is not expressed neither in glucose or xylose, strongly suggesting that these genes are not involved in the composition of xylose by the ALE-xyl strain.

### Assay of xylose reductase activity

To determine if the ALE-xyl cells growing in xylose express a xylose reductase, a crude extract of these cells was analysed to determine the presence of this activity. Figure S7 shows that xylose reductase activity is detectable in the crude extract, indicating that xylose can be converted into xylitol. Figure S7 also shows that as usual xylose reductase can utilize both NADH and NADPH as cofactors.

Interestingly, when the ALE-xyl crude extract was analysed by HPLC to detect the presence of xylitol we observed a pick in the chromatogram corresponding to a small amount xylitol (0.5 mg/mL of extract), which agrees with the expression of the xylitol reductase.

## Discussion

Although recombinant organisms can be designed *à la carte*, they do not always meet al.l the requirements for their industrial use. In addition, the use of GMOs (Genetically Modified Organisms) requires the utilization of containment procedures during the whole process even if they are classified as BSL-1. Moreover, when using plasmids for such metabolic engineering designs, antibiotics are usually required to maintain the plasmid stability. Consequently, in many cases the industry tends to use improved microorganisms that do not involve GMOs. One of the non-recombinant strategies that can be used to improve the efficiency of industrial microorganisms is ALE [22]. This adaptation occurs because of the redundancy of these microorganisms in genes/proteins/enzymes functions. Furthermore, genes mutate through natural mechanisms, changing their original functions or regulations, or phenotypic changes occur that only remain as far as the pressure persists [39, 40].

In this work we show that it is possible to evolve by ALE a relevant oleaginous microorganism, such as *R. jostii* RHA1, to confer it the ability of metabolizing xylose. The selection of this evolved strain of *R. jostii* RHA1 was relatively easy, probably due to the fact that this strain has a large genome (10 Mb) that enable the presence of many flexible phenotypes. Actually, after few weeks of culture in a minimal medium containing xylose, a significant amount of biomass was observed facilitating the isolation of the evolved ALE-xyl strain. However, considering that the capacity to metabolize xylose was due to a phenotypical adaptation, ALE-xyl cells must be always cultured in minimal medium containing xylose as sole carbon and energy source to keep the xyl phenotype.

Although the heterologous *xylA* and *xylB* genes are sufficient to confer the ability to metabolize xylose, the RHA1 recombinant strain should have other metabolic bottlenecks for an efficient consumption of xylose. Most likely these metabolic burdens are related to pentose transport, but we cannot rule out that other fine metabolic setting related to energy and redox balances are required to optimize xylose metabolism. Apparently, these fine-tuned changes can be more easily achieved by ALE since these settings usually depend of the acquisition of different mutations that can be accumulated and selected at the same time during the ALE process. Therefore, we assume that a recombinant strain like *R. jostii* RHA1 (pNVSxylABatf1) must be evolved by ALE to increase its xylose metabolic efficiency. In fact, the utilization of xylose has been improved by ALE in other engineered microorganisms [41, 42], such as, *Pseudomonas putida* S12 [11] or yeast strains [43, 44].

Probably the most interesting result of this work, is the fact that the ALE-xyl strain was able to metabolize at the same time glucose and xylose with no catabolic repression by glucose, and therefore, we have not observed a biphasic growth curve that might hinder the lipid production fermentation strategy when using saccharified lignocellulosic media. In this sense, Millán et al. (2020) used ALE to improve the assimilation of xylose and glucose in *Azotobacter vinelandii*, however, in this case, the co-utilization of both appears to be fundamental since xylose is poorly used in the absence of glucose [45]. Moreover, Jin et al. (2019) accelerated by ALE the simultaneous conversion rate of glucose and non-glucose sugars derived from lignocellulose biomass in *Gluconobacter oxydans*. Interestingly, this strain is naturally able to metabolize xylose and arabinose, but with low efficiency, demonstrating that even in a natural consumer a fine tuning of metabolism provides a further advantage [46]. In addition, the co-consumption of glucose and xylose has been also improved by ALE in yeast strains [47].

Remarkably, we have determined that the production of lipids in the ALE-xyl strain using xylose as carbon source is at least comparable to, or even superior than the production of lipids with glucose, suggesting that xylose metabolism couples perfectly to lipid biosynthesis in this strain.

Trying to understand how ALE-xyl strain has evolved to consume xylose we performed an in-silico analysis of *R. jostii* RHA1 genome, but it did not reveal the presence of a putative complete xylose metabolic pathway in this strain. In this sense [14], have demonstrated that *RHA1_ro02812* (*xylB1*) and *RHA1_ro02901* (*xylB2*) genes encode functional xylulokinases enzymes (XylB), but to metabolize xylose *R. jostii* RHA1 requires the cloning and expression of an heterologous xylose isomerase (XylA). Interestingly, Xiong et al. (2012) showed that the *R. jostii* RHA1 recombinant strain harbouring the heterologous XylA gene grows poorly and it must be evolved by ALE to improve its xylose consumption, suggesting that other genes are required to consume xylose efficiently [14].

The transcriptomic analysis revealed that the *pen* operon *RHA1_ro02898-RHA1_ro02909*, containing *xylB2* (*RHA1_ro02901*), was overexpressed in xylose. The *pen* operon also encodes an ABC pentose transporter that according to our hypothesis can facilitate the uptake of xylose, but it does not encode a putative xylose isomerase (XylA) that could allow us to propose the existence of a silent xylose isomerase pathway. Therefore, according to the gene expression levels in ALE-xyl (Fig. 6), we propose that xylose can be transformed into xylulose by a reductase pathway using a xylose reductase. In agreement with this hypothesis, we have detected a low but significant xylose reductase activity in the crude extract of ALE-xyl. Moreover, we have detected the presence of small amounts of xylitol in the crude extract indicating that xylose is transformed into xylitol in this evolved strain.

Interestingly, a similar finding that reinforces our hypothesis was observed by Sekar et al. (2016) when they evolved *S. oneidensis* to consume xylose [35]. They were able to activate a silent reductase xylose pathway of *S. oneidensis* after 12 months of ALE evolution demonstrating that a mutation in an MFS transporter that might facilitate the xylose uptake was crucial to metabolize xylose. Moreover, they also demonstrated that an aldolase/keto reductase has a xylose reductase activity and transform xylose into xylitol. Although, they could not detect a specific xylitol/xylulose dehydrogenase activity, they propose that there are many alcohol dehydrogenases that can fulfil this role [35]. Therefore, the key factor to un-silent the xylose metabolic pathway in *S. oneidensis* appears to be an efficiently xylose uptake as appears to be the case in ALE-xyl strain. This is, in the ALE-xyl strain, we have observed that the *RHA1_ro04589* gene that encodes a putative xylose reductase, and the *RHA1_ro02809* gene that encodes a putative xylitol dehydrogenase are highly expressed in RHA1 both in glucose and xylose and thus, the critical factor that can render ALE-xyl strain the capacity to consume xylose is the overexpression of the *penABC* genes that encode a pentose transporter. In this sense, although [35] were able to activate by ALE a silent xylose metabolic pathway of *S. oneidensis*, in contrast with ALE-xyl strain which grows very efficiently in xylose, the *S. oneidensis* strain had to be engineered to improve its growth on xylose [18]. Therefore, we speculate that the high efficiency to metabolize xylose in the ALE-xyl strain can be probably due to the high efficiency of the ABC transport system induced in this strain when compared to the mutated MSF transporter in *S. oneidensis*. Other studies have highlighted the crucial role of xylose transporters in preventing glucose catabolic repression, as demonstrated in *Ogataea polymorpha*, where enhanced xylose assimilation was achieved by introducing a hexose transporter (HXT1*) with higher affinity for xylose, allowing for efficient co-utilization of glucose and xylose in mixed-sugar conditions [48, 49].

Although the analysis of the genes encoded by the *pen* operon *RHA1_ro02898-RHA1_ro02909* does not allow to propose a clear function for this operon, the presence of putative pentose ABC transporter (*penABC*) and the *penHIJ* genes encoding the putative xylulokinase, phosphoglycolate phosphatase and ribose-5-phosphate isomerase (RipA) enzymes suggests its implication in a phosphorylating pentose pathway. A similar pentose metabolizing function of this operon in RHA1 has been proposed by Iino et al. (2012) [50].

In spite of the fact that we have detected the presence of xylitol in the ALE-xyl strain suggesting that xylose is consumed by a reductase pathway we cannot completely discard that PenJ (RipA), i.e., the second copy of a ribose 5-phosphate isomerase in RHA1, might transform xylose into xylulose, assuming a different role for this enzyme. In this sense, it has been demonstrated that ribose-5-phosphate isomerase (RipA) from *Ochrobactrum* can isomerize several non-phosphorylated pentoses [51]. Therefore, the second copies of RipA (PenJ), XylB (PenH) found in the *pen* operon of RHA1 together with the PenABC pentose transporter might be part of a new pentose metabolizing pathway. In this sense, it is worth to mention that *pen* operon contains other 5 genes, *penDEFGK*, that can be partially correlated by BLAST analyses with the genes found in non-phosphorylating pentose metabolizing pathways reinforcing the assumption that this operon is related to pentose metabolism [6, 52].

## Conclusions

This work shows that the oleaginous strain *R. jostii* RHA1 can acquire by ALE the ability to metabolize xylose without adding heterologous genes. Our results demonstrate that RHA1 contains silent capabilities to metabolize pentoses that can be unsilenced by adaptation to a xylose medium. The evolved ALE-xyl strain is no only able to efficiently use xylose as carbon source to grow and produce lipids, but also capable of using glucose and xylose at the same time to accumulate lipids with a high efficiency. Therefore, this evolved strain emerges as a promising microbial factory for industrial bio-oils production from saccharified lignocellulosic materials.

## Electronic supplementary material

Below is the link to the electronic supplementary material.

Supplementary Material 1

## Data Availability

Data is provided within the manuscript or supplementary information files.
