# Supplementary material for: Unsilencing a cryptic xylose metabolic pathway in Rhodococcus jostii RHA1 for efficient lipid production from lignocellulosic biomass
Source: J Biol Eng. 2025 Apr 14;19:32. doi: 10.1186/s13036-025-00503-1 (PMC11998424; doi:10.1186/s13036-025-00503-1)
Supplement: Supplementary file 1 — Supplementary Material 1 [file 13036_2025_503_MOESM1_ESM.docx]

**Appendix A. Supplementary data**

**Unsilencing a cryptic xylose metabolic pathway in Rhodococcus jostii RHA1 for efficient lipid production from lignocellulosic biomass**

**Miguel G. Acedos^a,b^, Isabel de la Torre^a^, Jorge Barriuso^a^, José L. García^a^**

^a^ Department of Plant and Microbial Biotechnology. Centro de Investigaciones Biológicas Margarita Salas. Consejo Superior de Investigaciones Científicas (CSIC). Madrid. Spain

^b^ Advanced Biofuels and Bioproducts Unit, Department of Energy. Centro de Investigaciones Energéticas, Medioambientales y Tecnológicas (CIEMAT). Madrid. Spain

Corresponding authors: M. G. Acedos & J. L. García. miguel.garciaacedos@ciemat.es & [jlgarcia@cib.csic.es](mailto:jlgarcia@cib.csic.es)

**Supplementary tables and figures**

**Table legends**

Table S1. Genes encoding the Entner-Doudoroff pathway of *R. jostii* RHA1

Table S2. Comparison of expression levels of ribosomal genes in xylose vs glucose.

**Figure legends**

Figure S1. Experimental design of ALE for evolving *R. jostii* RHA1.

Figure S2. A) Scheme of pNVs shuttle vector and the synthetic *xylABatf1* operon. B) sequence of the synthetic *xylABatf1* operon.

Figure S3. A) Heat map of gene expression profile under glucose and xylose growth conditions of *R. jostii* RHA1 ALE-xyl. The diagram shows the hierarchical clustering of genes, showing the log2 of the normalized mean expression of those in which FC is greater than 2 or less than -2. B) Principal component analysis (PCA) plot. The plot show gene expression pattern distances between replicates

Figure S4. Growth of wild type *R. jostii* RHA1, *R. jostii* RHA1 ALE-xyl adapted strain and *R. jostii* RHA1 (pNVSxylABatf1) on LB or LB plus kanamycin and W minimal medium with xylose or W minimal medium with xylose plus kanamycin.

Figure S5. Loss of phenotype of R. jostii RHA1 ALE-xyl strain to metabolize xylose in LB broth and minimal W broth with xylose. A) Picture of plates of each growth B) cfu/mL counts in each culture broth and run.

Figure S6. Phase contrast microscopy and fluorescence images of R. jostii RHA1 ALE-xyl and R. jostii RHA1 (pNVSxylAB) strains. In the fluorescence images, the lipid bodies inside the cells can be seen in green. Fluorescence intensity has not been standardized and cannot be used to quantify lipid content.

Figure S7. Enzymatic assays of xylose reductase activity using xylose as substrate and NADH and NADPH as cofactor. Solid lines represent the enzymatic activities measured in the presence of crude extracts, cofactor and substrate. Dashed lines represent the enzymatic activity measured in the absence of substrate as control.

Table S1. Expression levels of genes involved in the Entner-Doudoroff pathway

| Gene | Function | Counts in glucose | Counts in xylose |
| --- | --- | --- | --- |
| *RHA1_ro02362* | gluconokinase | 668 | 627 |
| *RHA1_ro02363* | gluconate:H+ symporter | 56 | 57 |
| *RHA1_ro02364* | FadR/GntR regulator | 74 | 104 |
| *RHA1_ro02365* | glucose porter family MFS transporter | 6005 | 3275 |
| *RHA1_ro02366* | ROK family transcriptional regulator | 1161 | 6703 |
| *RHA1_ro02367* | bifunctional 4-hydroxy-2-oxoglutarate aldolase/2-dehydro-3-deoxy-phosphogluconate aldolase | 3082 | 15962 |
| *RHA1_ro02368* | phosphogluconate dehydratase | 9086 | 60540 |
| *RHA1_ro02369* | glucose-6-phosphate dehydrogenase | 11372 | 64163 |
| *RHA1_ro02370* | ROK family protein (glucokinase or regulator) | 1022 | 5234 |

Table S2. Comparison of expression levels of ribosomal genes in xylose vs glucose.

| Locus | Name | Log2 fold-chain | P-value |
| --- | --- | --- | --- |
| *RHA1_ro06132* | 30S ribosomal protein S10 | 5.09 | 0 |
| *RHA1_ro06133* | 50S ribosomal protein L3 | 5.24 | 0 |
| *RHA1_ro06134* | 50S ribosomal protein L4 | 5.40 | 0 |
| *RHA1_ro06135* | 50S ribosomal protein L23 | 5.77 | 0 |
| *RHA1_ro06136* | 50S ribosomal protein L2 | 5.46 | 0 |
| *RHA1_ro06137* | 30S ribosomal protein S19 | 5.82 | 0 |
| *RHA1_ro06138* | 50S ribosomal protein L22 | 5.21 | 0 |
| *RHA1_ro06139* | 30S ribosomal protein S3 | 5.32 | 0 |
| *RHA1_ro06140* | 50S ribosomal protein L16 | 5.28 | 0 |
| *RHA1_ro06141* | 50S ribosomal protein L29 | 5.02 | 7.40E-238 |
| *RHA1_ro06142* | 30S ribosomal protein S17 | 5.05 | 0 |
| *RHA1_ro06143* | 50S ribosomal protein L14 | 3.71 | 3.77E-289 |
| *RHA1_ro06144* | 50S ribosomal protein L24 | 3.79 | 1.60E-224 |
| *RHA1_ro06145* | 50S ribosomal protein L5 | 3.79 | 1.78E-249 |
| *RHA1_ro06146* | type Z 30S ribosomal protein S14 | 3.97 | 4.26E-118 |
| *RHA1_ro06147* | 30S ribosomal protein S8 | 3.58 | 0 |
| *RHA1_ro06148* | 50S ribosomal protein L6 | 3.41 | 3.24E-264 |
| *RHA1_ro06149* | 50S ribosomal protein L18 | 2.99 | 4.53E-125 |
| *RHA1_ro06150* | 30S ribosomal protein S5 | 3.21 | 3.33E-223 |
| *RHA1_ro06151* | 50S ribosomal protein L30 | 2.67 | 5.87E-48 |
| *RHA1_ro06152* | 50S ribosomal protein L15 | 2.55 | 2.44E-155 |
| *RHA1_ro06153* | preprotein translocase subunit SecY | 2.12 | 2.63E-92 |
| *RHA1_ro06154* | adenylate kinase | 1.28 | 1.38E-20 |
| *RHA1_ro06155* | type I methionyl aminopeptidase | 1.28 | 1.56E-20 |
| *RHA1_ro06156* | D-aminoacyl-tRNA deacylase | 1.49 | 7.49E-09 |
| *RHA1_ro06157* | translation initiation factor IF-1 | 3.0 | 1.48E-135 |
| *RHA1_ro06158* | 50S ribosomal protein L36 | 3.31 | 9.44E-230 |
| *RHA1_ro06159* | 30S ribosomal protein S13 | 3.49 | 6.57E-147 |
| *RHA1_ro06160* | 30S ribosomal protein S11 | 3.03 | 7.44E-142 |
| *RHA1_ro06161* | 30S ribosomal protein S4 | 2.58 | 1.00E-118 |
| *RHA1_ro06162* | DNA-directed RNA polymerase subunit alpha | 2.25 | 6.41E-95 |
| *RHA1_ro06163* | 50S ribosomal protein L17 | 1.62 | 4.75E-53 |
| *RHA1_ro06164* | tRNA pseudouridine(38-40) synthase TruA | 1.80 | 3.18E-20 |
| *RHA1_ro061171* | 50S ribosomal protein L13 | 4.03 | 0 |
| *RHA1_ro061172* | 30S ribosomal protein S9 | 4.63 | 0 |


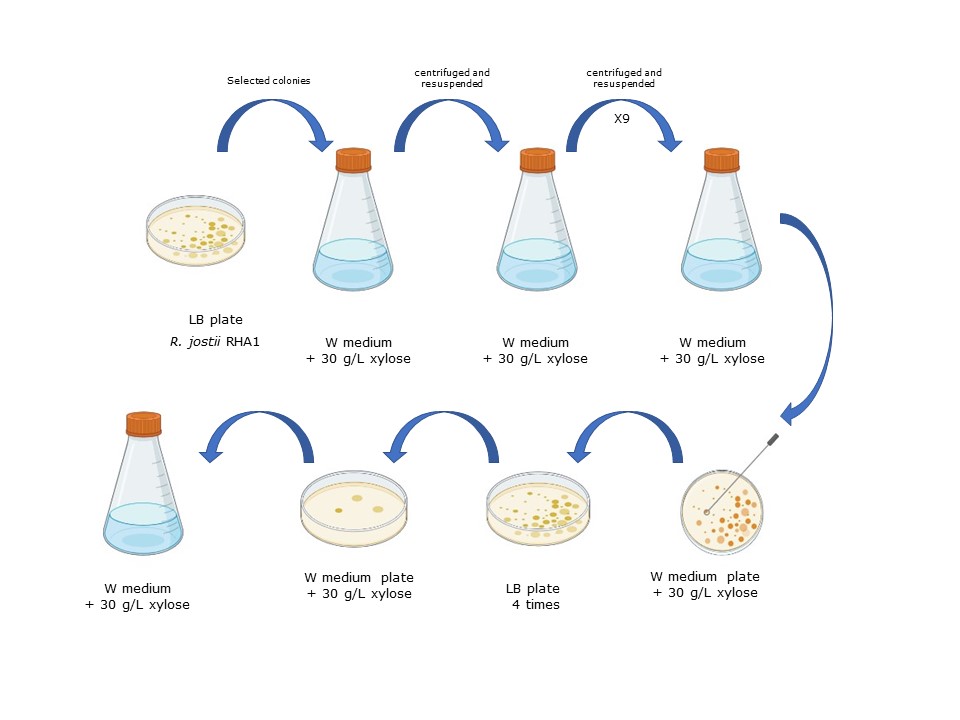


Figure S1

**A)**


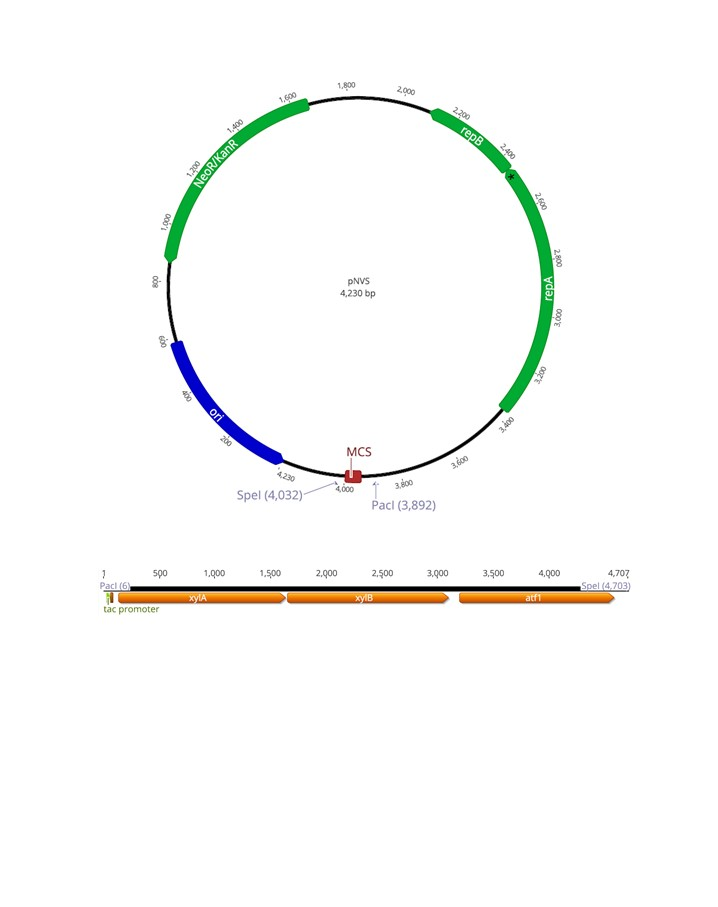


**B)**

TTAATTAAGGGATGGTACCCTGAGCTGTTGACAATTAATCATCGGCTCGTATAATGTGTGGAATTGTGAGCGGATAACAATTTCACACAGGAAACACAATTCATCGATAGTACTCGGCAAGGAGATCAACAAATGACCATCTTCGACAACTACGAAGTGTGGTTCGTCATCGGTTCGCAGCACCTGTACGGCCCCGAGACGCTGCGACAGGTCACGCAGCATGCCGAACACGTGGTGAATGCGCTCAACACCGAGGCGAAGCTCCCCTGTAAGCTGGTGCTCAAGCCCCTGGGGACCACACCGGATGAGATCACCGCCATATGCCGCGACGCCAACTACGACGACCGCTGCGCCGGTCTCGTAGTCTGGCTGCACACCTTCTCGCCGGCGAAGATGTGGATCAACGGCCTTACCATGCTCAACAAGCCGCTGCTCCAGTTCCATACACAATTCAATGCAGCTTTGCCGTGGGACTCGATCGACATGGACTTCATGAACCTCAACCAGACCGCCCACGGTGGGCGTGAGTTCGGCTTCATTGGCGCCCGGATGCGTCAGCAGCACGCGGTCGTCACGGGTCACTGGCAGGACAAGCAGGCGCACGAGCGGATCGGTTCATGGATGCGGCAAGCGGTTAGCAAGCAAGACACCCGCCACCTGAAGGTATGCCGGTTCGGCGACAACATGCGCGAGGTCGCAGTCACCGACGGCGACAAAGTCGCCGCGCAGATCAAGTTCGGGTTTTCCGTTAACACCTGGGCCGTCGGCGATTTGGTGCAGGTGGTCAACTCGATCTCCGACGGAGACGTGAACGCGCTCGTCGACGAGTATGAATCGTGCTACACGATGACGCCCGCCACCCAGATCCACGGGAAGAAGCGCCAGAACGTCCTCGAAGCCGCCCGCATCGAACTGGGCATGAAGCGCTTCCTGGAGCAGGGCGGGTTCCACGCGTTCACCACCACGTTCGAGGACCTGCACGGCTTGAAGCAGCTGCCGGGCCTGGCAGTGCAGCGGCTGATGCAGCAGGGGTACGGATTCGCCGGCGAGGGCGACTGGAAGACCGCGGCGCTCTTGCGGATCATGAAAGTGATGTCCACGGGTCTCCAGGGCGGTACTTCCTTCATGGAGGACTACACCTACCACTTCGAGAAGGGCAACGACCTGGTCCTGGGCTCGCACATGCTCGAGGTCTGCCCGAGCATCGCCGCAGAGGAGAAGCCGATCCTGGATGTCCAGCACCTCGGGATCGGAGGCAAGGACGACCCGGCGCGCCTCATCTTCAACACGCAGACGGGACCCGCGATCGTGGCCTCCCTGATCGATCTCGGCGACCGCTACCGGCTCCTCGTCAACTGCATCGACACCGTCAAAACGCCGCACAGCCTCCCGAAGCTCCCGGTGGCGAACGCGCTGTGGAAGGCCCAGCCCGACCTCCCCACCGCCTCGGAAGCGTGGATCTTGGCAGGCGGTGCGCACCACACCGTGTTCAGCCATGCCCTAAACCTGAACGACATGCGGCAGTTCGCCGAGATGCACGACATCGAGATCACGGTGATCGACAATGATACCCGACTGCCAGCGTTCAAGGACGCGCTCCGGTGGAACGAGGTCTACTACGGCTTCCGCAGGTAACGGAGAGGGAGAAGCCCGATGAGCGCGGCGGAGGGCCCTCTGGTCGTCGGAGTGGACACCAGCACCCAGTCGACCAAAGCCCTCGTGGTCGACGCCGCCACCGGCCGCGTGGTGGCCTCGGGACAGGCGCCCCACACCGTCTCGTCGGGCACCGGCCGGGAGTCCGACCCGCGGCAGTGGTGGGACGCGCTCGGTGAGGCCCTGTCGCAGTGCGGCGAAGCTGCCCGCGAAGCGGCAGCAGTTTCCGTCGGCGGGCAACAGCACGGCCTGGTGACGCTCGACGCCCGAGGCGAACCGGTCCGCCCCGCGTTGCTGTGGAACGACGTCCGTTCGGCGCCACAGGCCCGACGGCTGATCGACGAACTGGGCGGGGCGAAGGCGTGGGCCGAGCGCACCGGCAGCGTCCCGTCCGCGTCCTTCACCGTCACGAAGTGGGCGTGGCTGACCGAGCACGAGCCGGAGGCCGCCCGCGCCGTGAAGGCAGTCCGGCTCCCGCACGACTACCTGACCGAACGCCTCACGGGAGAGGGCACCACCGACCGTGGCGACGTGTCCGGCACGGGCTGGTGGGCGTCCGGCACTGAGGCGTACGACGAGGAGATCCTCGCCCGGGTCGCCCTGGACCCGGCCCTGCTGCCCCGCGTCGTGCGGCCCGGCGAGGTCGCCGGTACGGTGCGCGACGGCCACGGTCTCCCGTTCTCGAAGGGCACTCTCGTCGCAGCGGGCACGGGTGACAACGCCGCGGCCGCCTTGGGTCTGGGGCTGCGCCCGGGCGTGCCCGTGATGTCCCTCGGGACGAGTGGAACCGCGTACGCCGTTAGCCAGCGCCGTCCCGCCGATCCCACCGGTACAGTAGCGGGGTTCGCCGACGCACGCGGGGACTGGCTCCCGCTCGCGTGCACCCTCAACTGCACGCTAGCGGTCGACCGGGTGGCGTCGCTGCTCGGACTCGACCGCGAGGCCGTCGAACCCGGCACGGACGTGACCCTGCTCCCGTTCCTCGACGGGGAACGGACCCCGAACCTGCCGCATTCCTCGGGGCTCCTGCACGGGCTGCGGCACGACACAACCGCCGGCCAGCTGCTCCAGGCGGCCTACGACGGCGCGGTGCACAGCCTCCTTGGAGCGTTGGATCTCGTCCTCGACGCCGACGCCGACCCCTCGGCGCCCCTCCTCCTCATCGGCGGCGGCGCCCGCGGCACGGCCTGGCAGCAGACCGTGCGCCGCTTGAGCGGACGGCCGGTGCAGATCCCGGACGCGCGGGAACTCGTCGCACTGGGTGCGGCGGCGCAGGCGGCCGGGCTGCTGACGGGCGAGGACGCCGCCGCGGTCGCTAGGCGGTGGAATACCGCCGCCGGGCCCGTCCTGGATGCCGTCGAGCGGGACGAGGCGACGCTGAACCGAATCACCGGTGTGCTGTCAGATGCGGCACCGCTGCTCGAGCGGGATGCTGCGAGTCGCTAACGGAGGTACCACGCGGATCCGCGTACCCGGAATTCCGGGACCAAAGCTTGGGTCCAACTGCAGAACCAATGCATTGGGGATAGAGGGAGAAGCCCGATGACCCAGACCGATTTCATGAGCTGGCGGATGGAGGAGGACCCAATCCTCCGCTCGACGATCGTGGCGGTCGCGTTGCTGGATCGTTCCCCCGACCAGAGCCGCTTCGTCGACATGATGCGCCGGGCCGTGGACCTGGTCCCGCTCTTCCGGCGCACCGCCATCGAGGCACCGATGGGCTTCGCGCCGCCGCGCTGGGCGGACGACCACGACTTCGACCTGTCGTGGCACCTCCGGCGATATACCCTCCCGGAACCGCGGACCTGGGACGGCGTTCTGGATTTTGCGCGCACGGCCGAGATGACCGCATTCGACAAGCGTCGCCCCCTGTGGGAGTTCACCGTGCTCGACGGCCTGCACGACGGGCGGTCCGCGCTCGTGATGAAGGTCCACCACAGCCTGACCGATGGTGTGTCCGGCATGCAGATCGCCCGCGAGATCGTCGACTTCACGCGCGACGGCGGTCCTCGTCCCGACCGGACCGACCATCGTACGGCGGCCCCGAACGGCAAGTCGCCGACGCCCCGGGGAAGACTGTCCTGGTATCGCAACTCCGCTACGGACGTGGCGCGGCGAGCGTCCAACACCCTCGGACGGAATAGCGTGCGGCTCGTCCGCACGCCGCGCGCCACGTGGCGTGACGCGGCGGCACTCGCTGGTTCCACGCTGCGCCTCACCCGGCCCGTCGTCTCGACGTTGTCGCCCGTCATGAAGAAACGGTCGACAAGGCGGCACTGCGCCGTCCTCGACGTCCCGGTCGAGGCGCTGGCGCAAGCAGCCGCAGCGGGAGCCGGCAGCATCAACGACGCCTTCCTGGCCGCGGTCTTGCTCGGGATGGCGAAGTACCACCGACTGCACGGCGCGGAAATCTCCGAACTCCGCATGACGCTGCCGATTTCGCTGCGCGCCGAGACCGATCCGGTAGGCGGCAACAGGATCACCCTGGCCCGGTTCGCCCTGCCGGCCGACATCGACGACCCCGCCGAACTAATGCACCGGGTGCATGCGACGGTCGACGCCTGGCGCCACGAGCCGGCGATCCCCCTCTCGCCCACCATCGCGGGGGCCCTGAACCTCCTCCCGGCGTCCACCCTCGGCAACATGCTCAAGCAGCGCGCGTTCGTGGCCTCGAACGTCGTGGGGTCGCCGGTGCCGCTGTTCATCGCCGGATCTGAGGTGCTGCACTACTACGCCTTCAGCCCCACCCTGGGGTCAGCGTTCAACGTGACCCTGATGTCGTACACGACCCGCTGCTGCGTCGGCATCAACGCCGACACGGACGCCATCCCGGACCTCGCGACCCTTACAGACTCGATCGCCGACGGTTTCCGCGCCGTCCTAGGCCTCTGTACCAAGACCACCGACACCCGCGTGGTGGTTGCCAGTTAAATTTGTCCTACTCAGGAGAGCGTTCACCGACAAACAACAGATAAAACGAAAGGCCCAGTCTTTCGACTGAGCCTTTCGTTTTATTTGCGAACTGCAGAACCAATGCATTGGTTGTGGACTAGT.

Figure S2


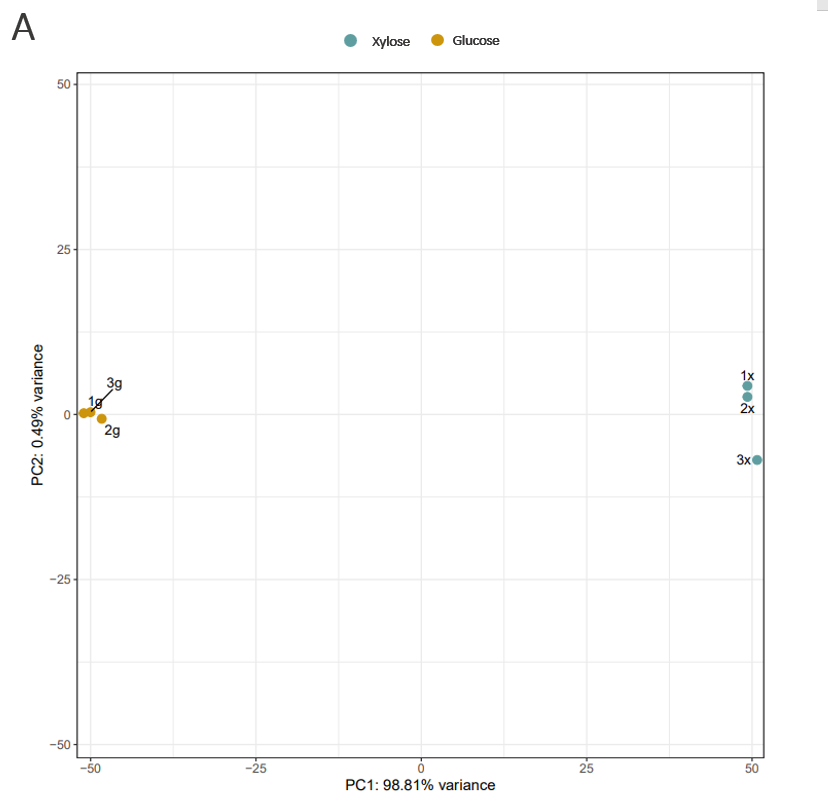


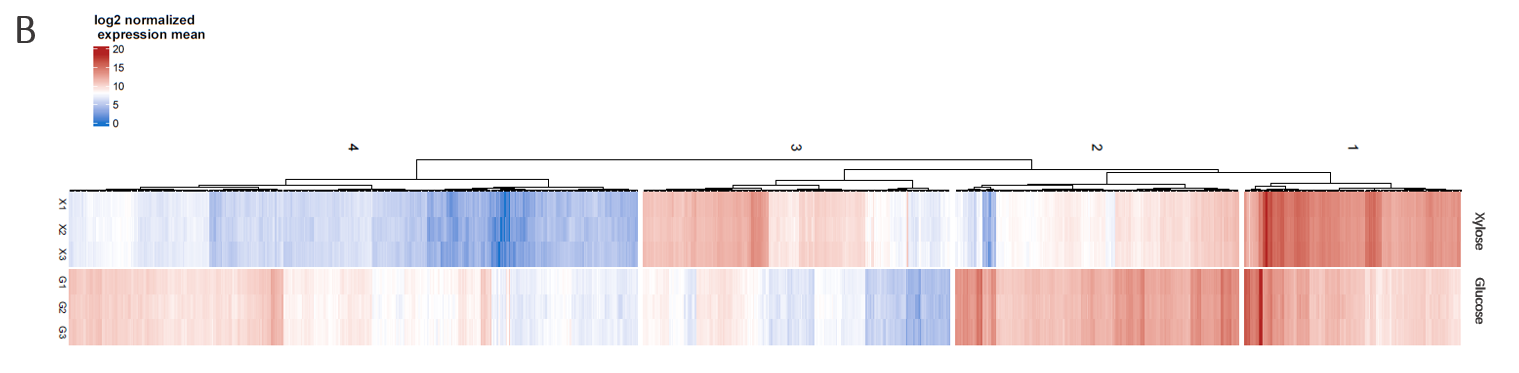


Figure S3


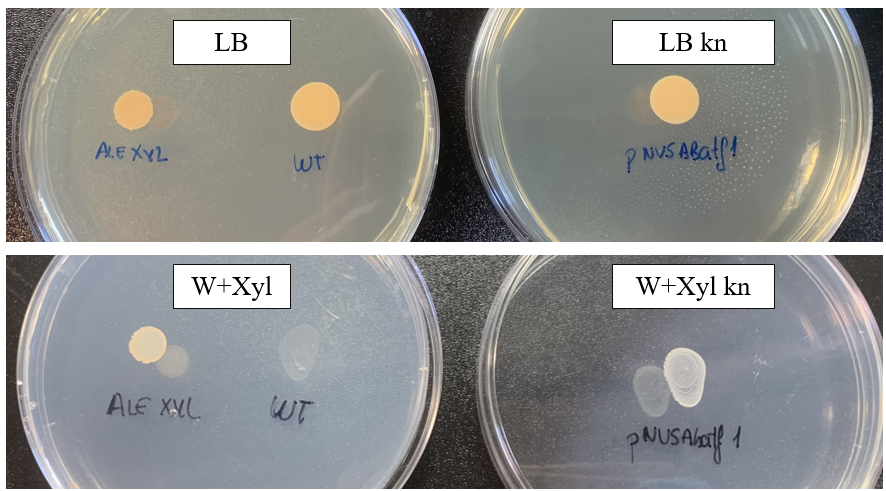


Figure S4


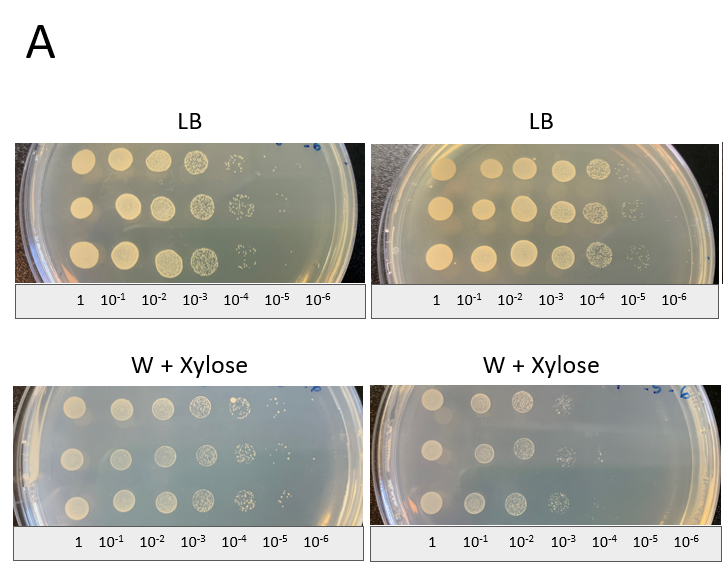


Figure S5

| Strain | Carbon source | Microscope FC | Fluorescence |
| --- | --- | --- | --- |
| *R. jostii* RHA1 (pNVsXylABatf1) | Xylose | 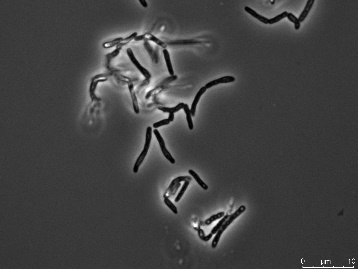 | 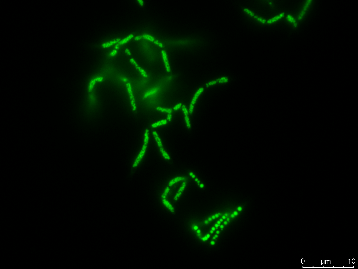 |
| *R. jostii* RHA1 ALE-xyl | Xylose | 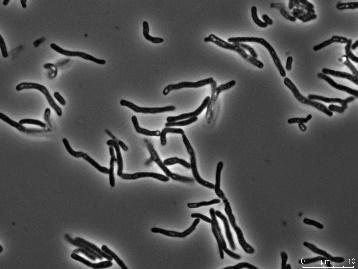 | 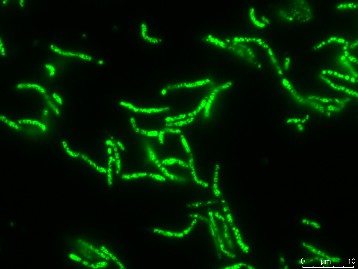 |

Figure S6.

Figure S7.
